# COVID-19 severe acute respiratory syndrome in Brazilian newborns in 2020-2021

**DOI:** 10.1590/1980-549720230012

**Published:** 2023-02-20

**Authors:** Andressa Rocha Pereira, Maria dos Remédios Freitas Carvalho Branco, Silmery da Silva Brito Costa, Denise Ailine Monteiro Lopes, Vanessa Vieira Pinheiro, Daniel Cavalcante de Oliveira, Amanda Namíbia Pereira Pasklan, Jamesson Amaral Gomes, Alcione Miranda dos Santos, Mônica Elinor Alves Gama

**Affiliations:** I Universidade Federal do Maranhão, School of Medicine – São Luís (MA), Brazil.; II Universidade Federal do Maranhão, Graduate Program in Collective Health – São Luís (MA), Brazil.; III Universidade Federal do Ceará, Campus Santa Casa de Misericórdia de Fortaleza – Fortaleza (CE), Brazil.; IV Universidade Federal do ABC, Graduate Program in Biomedical Engineering – São Bernardo do Campo (SP), Brazil.; V Universidade Federal do Maranhão, School of Medicine – Pinheiro (MA), Brazil.; VI Department of Public Safety – São Luís (MA), Brazil.

**Keywords:** COVID-19, Severe acute respiratory syndrome, Newborn, Brazil, Epidemiology, Public health, COVID-19, Síndrome respiratória aguda grave, Recém-nascido, Brasil, Epidemiologia, Saúde pública

## Abstract

**Objective:**

To describe the clinical characteristics of cases of COVID-19 severe acute respiratory syndrome (SARS) in Brazilian newborns (NBs) in 2020 and 2021, recorded in the Influenza Epidemiological Surveillance Information System (*Sistema de Informação da Vigilância Epidemiológica da Gripe* — SIVEP-Gripe).

**Methods:**

The variables analyzed were gender, race/skin color, hospitalization, intensive care unit (ICU) admission, use of ventilatory support, signs and symptoms (fever, cough, O_2_ saturation<95%, dyspnea, respiratory distress, diarrhea, and vomiting), progress (death or cure), risk factors/comorbidities. Categorical variables were expressed as absolute and relative frequencies.

**Results:**

We found 1,649 records of COVID-19 SARS in NBs, with a predominance of multiracial babies in both years. The most frequent symptoms in 2020 and 2021 were, respectively: respiratory distress (67.0 and 69.7%), fever (46.3 and 46.2%), and cough (37.0 and 46.3%). In 2020, 30.5% of patients received invasive ventilatory support; in 2021, this number was 41.6%. In addition, more than 55% of cases required ICU admission, and over 16% died.

**Conclusion:**

We emphasize the high proportion of cases that required intensive care and progressed to death.

## RESUMO

**Objetivo:**

Descrever as características clínicas dos casos de Síndrome Respiratória Aguda Grave (SRAG) por COVID-19 em recém-nascidos (RNs) em 2020 e 2021, no Brasil, registrados no Sistema de Informação da Vigilância Epidemiológica da Gripe (SIVEP-Gripe).

**Métodos:**

As variáveis analisadas foram: sexo, raça/cor, hospitalização, internação em unidade de terapia intensiva (UTI), uso de suporte ventilatório, sinais e sintomas (febre, tosse, saturação de O_2_<95%, dispneia, desconforto respiratório, diarreia e vômitos), evolução (óbito ou cura), fatores de risco/comorbidades. As variáveis categóricas foram apresentadas em frequências absolutas e relativas.

**Resultados:**

Houve 1.649 registros de SRAG por COVID-19 em RNs, com predomínio de pardos nos dois anos. Os sintomas mais frequentes foram, respectivamente, em 2020 e 2021: desconforto respiratório (67,0 e 69,7%), febre (46,3 e 46,2%) e tosse (37,0 e 46,3%). Em 2020, 30,5% dos pacientes receberam suporte ventilatório invasivo, e 41,6% em 2021. Além disso, mais de 55% dos casos precisaram de internação em UTI, e acima de 16% morreram.

**Conclusão:**

Destaca-se a elevada proporção de casos que precisou de cuidados intensivos e que evoluíram para óbito.

## INTRODUCTION

In March 2020, the World Health Organization declared that COVID-19, a disease caused by the novel coronavirus identified as SARS-CoV-2, reached a pandemic state^1^. Although initially limited to older age groups, severe cases were progressively reported among neonates, posing great challenges to health care for this age range^2^.

Newborns (NBs) are considered a risk group due to the immaturity of their immune system and other conditions — such as prematurity — responsible for important changes in the respiratory system^3^. Thus, this research aimed to describe the clinical characteristics of cases of COVID-19 severe acute respiratory syndrome (SARS) in the age group from 0 to 28 days in Brazil in 2020 and 2021.

## METHODS

This is a descriptive study of COVID-19 SARS cases in the age group from 0 to 28 days in Brazil in 2020 and 2021, recorded in the Influenza Epidemiological Surveillance Information System (*Sistema de Informação da Vigilância Epidemiológica da Gripe* — SIVEP-Gripe), an official system for the record of SARS cases and deaths in the country.

The Ministry of Health has been monitoring SARS since the influenza A (H1N1) pandemic in 2009. Since then, SARS surveillance has been implemented in the Influenza and other respiratory viruses surveillance network, into which COVID-19 SARS was integrated in 2020^4^. Cases are reported to the “Individual Report Form for SARS Cases” (https://opendatasus.saude.gov.br/dataset/61d8e424-a008-47f3-80e1-2369f2f48cef/resource/25b6d4e6-31a8-4352-93e2-c9626bb529bc/download/ficha-srag-final-27.07.2020_final.pdf), which considers both hospitalized SARS patients and SARS deaths, regardless of hospitalization.

The study population was all NB cases (0 to 28 days of age) with a final classification of COVID-19 SARS, considering laboratory (antigen tests, polymerase chain reaction), imaging (X-ray and chest computed tomography), clinical (signs and symptoms), and epidemiological criteria (https://opendatasus.saude.gov.br/dataset/61d8e424-a008-47f3-80e1-2369f2f48cef/resource/9f6ba348-0033-49b1-abbe-719a0ffbeb28/download/dicionario-de-dados-srag-hospitalizado-27.07.2020-final.pdf). Data were collected from the portal https://opendatasus.saude.gov.br/dataset on March 9, 2022.

We used the following variables of the “Individual Report Form for SARS Cases”: gender (female or male); race/skin color (declared by the patient or guardian and following the categories of the Brazilian Institute of Geography and Statistics (*Instituto Brasileiro de Geografia e Estatística* — IBGE): White, Black, Asian, multiracial, and Indigenous); hospitalization (yes or no); ICU admission (yes or no); use of ventilatory support (yes, invasive support; yes, non-invasive support; or no); signs and symptoms (fever, cough, O_2_ saturation<95%, dyspnea, respiratory distress, diarrhea, and vomiting); case progress (cure or death); risk factors/comorbidities (yes or no): heart diseases, immunodeficiencies, neuropathies, lung diseases, Down syndrome, and others.

Prematurity — gestational age less than 37 weeks at birth — is not an option in the “risk factor” variable of the report form. When present, it is described in the “other risk factors/comorbidities” field.

Categorical variables were expressed as absolute and relative frequencies. We calculated absolute and relative frequencies of prematurity separately when it was specified in other risk factors/comorbidities.

Regarding the “progress” variable, the proportion of hospital deaths from COVID-19 SARS in the country was calculated by the ratio between the number of deaths in the age group from 0 to 28 days and the number of records in the SIVEP-Gripe per year of occurrence, multiplied by 100. Ignored items of all analyzed variables were excluded and considered losses. Analyses were performed in the Stata® software, version 16.

The Research Ethics Committee (REC) of the Hospital Universitário da Universidade Federal do Maranhão (HUUFMA) approved this study, under opinion number: 4,098,427 and CAAE 32206620.0.0000.5086, on June 19, 2020.

## RESULTS

In 2020, SIVEP-Gripe had 1,200,044 SARS cases recorded in Brazil, 712,299 of which were due to COVID-19. Among them, 793 were in the age group from 0 to 28 days. In 2021, this number reached 1,721,489 SARS cases, of which 1,189,433 were due to COVID-19; 856 in the age group from 0 to 28 days, totaling 1,649 COVID-19 SARS cases in NBs in two years.

In 2020, 51.3% were female; in 2021, 53.2% were male. We found a predominance of multiracial babies (64.0% in 2020 and 55.5% in 2021), followed by White NBs (30.3% in 2020 and 19.4% in 2021). The most frequent symptoms in 2020 and 2021 were, respectively: respiratory distress (67.0 and 69.7%), fever (46.3 and 46.2%), and cough (37.0 and 46.3%) (Table 1).

**Table 1:** Absolute and relative frequency of signs and symptoms, risk factors/comorbidities, intensive care unit admission, ventilatory support, and proportion of hospital deaths in newborns with COVID-19 severe acute respiratory syndrome, Brazil, 2020–2021.

Signs and symptoms	2020 n (%)	2021 n (%)
Fever	286/618 (46.3)	286/619 (46.2)
Cough	217/586 (37.0)	288/622 (46.3)
Dyspnea/Respiratory distress	431/643 (67.0)	476/683 (69.7)
Oxygen saturation<95%	250/573 (43.6)	324/622 (52.1)
Diarrhea	49/523 (9.4)	59/547 (10.8)
Vomiting	43/517 (8.3)	38/534 (7.1)
**Risk factors/Comorbidities**	**2020 n (%)**	**2021 n (%)**
Heart diseases	41/175 (23.4)	37/136 (27.2)
Immunodeficiencies	14/165 (8.5)	7/123 (5.7)
Neuropathies	11/165 (6.7)	7/123 (5.7)
Lung diseases	7/162 (4.3)	6/122 (4.9)
Down syndrome	5/161 (3.1)	1/120 (0.8)
Prematurity	3/242 (1.2)	11/197 (5.6)
Other	212/242 (87.6)	171/197 (86.8)
**Variables**	**2020 n (%)**	**2021 n (%)**
ICU admission	355/639 (55.6)	398/712 (55.9)
Invasive ventilatory support	130/617 (21.1)	157/688 (22.8)
Non-invasive ventilatory support	188/617 (30.5)	286/688 (41.6)
Proportion of hospital deaths	110/653 (16.8)	116/706 (16.4)

ICU: intensive care unit.

In 2020 and 2021, the proportion of ICU admissions, use of invasive ventilatory support, and hospital deaths was, respectively, 55.6 and 55.9%; 21.1 and 22.8%; 16.8 and 16.4% (Table 1). Heart disease was the most frequent comorbidity in both years; prematurity was reported in 1.2% of cases in 2020 and 5.6% in 2021 (Table 1). Of the cases that progressed to death, more than 70% required ICU admission, and more than 50% needed invasive ventilatory support (Table 2).

**Table 2:** Absolute and relative frequency of intensive care unit admission, use of ventilatory support, prematurity, and heart diseases in newborns who progressed to death from COVID-19 severe acute respiratory syndrome, Brazil, 2020–2021.

Variables	Year	
	2020 n (%)	2021 n (%)
ICU admission	60/75 (80.0)	55/74 (74.3)
Invasive ventilatory support	46/76 (60.5)	47/82 (57.3)
Non-invasive ventilatory support	17/76 (22.4)	17/82 (20.7)
Prematurity	2/110 (1.8)	2/116 (1.7)
Heart diseases	06/32 (18.7)	13/29 (44.8)

ICU: intensive care unit.

## DISCUSSION

SIVEP-Gripe had 1,649 COVID-19 SARS cases recorded in Brazilian NBs in 2020 and 2021. To date, the national and international literature has no analyses for the neonatal age group^5–7^ with numbers as significant as those presented in this study.

We found a predominance of multiracial babies, with a frequency greater than their proportion in the Brazilian population. In the United Kingdom, a study showed that neonates belonging to ethnic groups, including Black and multiracial NBs, accounted for half of the cases^5^. In England, children who were Black, multiracial, or belonged to other races presented a higher risk of hospitalization and longer length of stay compared to White children^8^.

Among the neonates’ symptoms analyzed in this study, the most prevalent ones were respiratory distress/dyspnea, fever, and cough. In a United Kingdom study of 66 neonates, the most common symptoms were fever, refusal to eat/lack of appetite, and vomiting, compared to older children and adults^5^. In a European study, fever, upper and lower respiratory tract symptoms, and gastrointestinal symptoms were more frequent among children and adolescents aged 0 to 18 years, in that order^9^.

Compared to adults, children generally have less severe clinical manifestations. A study of 60,109 patients in different age groups hospitalized for COVID-19 in 43 countries showed that symptoms such as fever, cough, and dyspnea are less prevalent in children under 18 years than in other age ranges^10^.

The proportion of ICU admission and hospital deaths revealed that neonates present severe forms of COVID-19. The immaturity of the innate and adaptive immune system in NBs could justify the greater severity of the disease in this age group, as the nature of the immune response at the beginning of life influences the susceptibility to immune-mediated diseases such as COVID-19^11^. Furthermore, international studies show that the age group under one year can be a risk factor for disease severity and consequent mortality among children^12,13^. A Chinese study with more than 2,135 COVID-19 cases in children and adolescents up to 19 years showed that children under one year had a higher proportion of severe disease (10.6%) compared to other age groups^12^.

This research identified higher proportions of ICU admission, use of invasive ventilation, and hospital mortality than studies conducted in other countries. In the United Kingdom, 42% of the 66 neonates analyzed had a severe infection, 36% received intensive care or ventilatory support, and none died^5^. Compared to other age groups, these proportions are even higher. In China, 6% of children and adolescents up to 18 years had severe disease^12^; in Europe, 8% required intensive care, and 4% needed mechanical ventilation^9^. In the USA, for example, from March 2020 to June 2021, the ICU admission rate of COVID-19 in the age group from 0 to 17 years was 26.5%, 6.1% required invasive ventilation, and 0.7% progressed to death^14^.

Not all patients who died were admitted to the ICU, probably due to a lack of available beds. Countries such as Brazil report higher pediatric COVID-19 mortality than high-income countries, possibly due to their inability to provide the necessary care for the most severe patients^15^. In Brazil, the significant racial, geographic, and socioeconomic disparities are factors admittedly associated with the quality of health care, and the recent experience with the SARS-CoV-2 coronavirus showed that the response capacity of the health service network decisively affects the case fatality rate of the disease, especially with respect to early case detection and the availability and access to critical care^16^.

In the country, the difficult access to health services and ICU beds can influence the severity of COVID-19 in this age group due to the greater need for hospitalizations and intensive care^17^, especially in the North and Northeast regions, which have the worst socioeconomic conditions and health indicators and the lowest proportions of pediatric ICU beds^3^. In this context, besides the deleterious effects of the disease, the worsening of the condition should be considered a consequence of the deficient care provided.

The most common risk factors/comorbidities were heart diseases and immunodeficiencies. Children with comorbidities are more likely to develop more severe forms of COVID-19 and have higher proportions of hospitalization^18^. Comorbidities/pre-existing medical conditions are associated with the development of more severe forms of the disease, especially chronic lung diseases^9^.

Prematurity represents another risk factor for more severe forms of COVID-19 and worse respiratory outcomes, particularly in children under 2 years^18^. Premature neonates have proportionally higher rates of SARS-CoV-2 infection^2^. However, in this study, prematurity was reported in only 14 RNs with COVID-19 SARS. Possibly because the “Individual Report Form for Hospitalized SARS Cases” has no specific field for prematurity. When present, it needs to be entered into the “Other” field.

The study limitations include the loss of information in some variables and failure to fill the race/skin color, risk factors, and case progress fields. In addition, this study has a descriptive and cross-sectional design, making it impossible to analyze the causal association between exposure and clinical outcomes. The strength of this work is the expressive number of COVID-19 SARS cases in NBs over 21 months across the country.

This study contributed to the literature by describing the clinical characteristics of COVID-19 SARS cases in Brazilian NBs. We suggest the performance of studies aimed at the COVID-19 scenario for NBs, especially regarding severe cases and unfavorable outcomes with deaths. We expect this study to add to the reflection on the importance of appropriate actions to prevent COVID-19 mortality in NBs.

Considering its possible underreporting, we recommend the inclusion of a specific field for prematurity as a risk factor in the “Individual Report Form for Hospitalized SARS Cases”. The need for a more even distribution of neonatal ICU beds in the country becomes evident, as they are concentrated in certain municipalities, hindering the access of NBs with severe cases to proper treatment^3^.
